# Detection of Aflatoxin B1-producing *Aspergillus flavus* strains from pistachio orchards soil in Iran by multiplex polymerase chain reaction method

**DOI:** 10.22034/CMM.2023.345055.1420

**Published:** 2023-09

**Authors:** Amin Daliri, Masoomeh Shams-Ghahfarokhi, Mehdi Razzaghi-Abyaneh

**Affiliations:** 1 Department of Mycology, Faculty of Medical Sciences, Tarbiat Modares University, Tehran, Iran; 2 Department of Mycology, Pasteur Institute of Iran, Tehran, Iran

**Keywords:** *Aspergillus flavus*, Aflatoxin B_1_, Multiplex-PCR, Pistachio orchards

## Abstract

**Background and Purpose:**

The current study aimed to report a multiplex polymerase chain reaction (PCR) assay as a monitoring technique to differentiate aflatoxigenic from non-aflatoxigenic strains of *Aspergillus flavus* isolated from pistachio orchards soil.

**Materials and Methods:**

In total, 25 *A. flavus* strains were isolated from soil samples of pistachio orchards. To test the strains for Aflatoxin B_1_ (AFB_1_)-producing ability, thin-layer chromatography (TLC) was used and the amounts of AFB_1_ were measured by high-performance liquid chromatography (HPLC). Multiplex PCR was used as a genome-based method to detect genes responsible for AFB_1_ production by *A. flavus* and the results were analyzed in terms of speed and specificity of detection. A set of four primers was designed specifically for the *omtA*, *omtB*, *ver-1*, and *aflR* genes which are commonly present in aflatoxin biosynthetic pathways.

**Results:**

The AFB_1_ production by the *A. flavus* strains ranged from 0 to 321 ρg/μl. Four-band patterns of the primer sets were observed only in AFB_1_-producing *A. flavus* strains. Moreover, 18 out of the 25 strains showed all four bands belonging to *omtA*, *omtB*, *ver-1*, and *aflR*, whereas 7 strains did not display *omtA*, or *aflR*-related bands, in non-toxigenic and low toxin-producing *A. flavus*.

**Conclusion:**

The multiplex PCR is a supplementary strategy to current conventional mycotoxin analytical techniques, such as TLC and HPLC. It could be used as an efficient method to differentiate aflatoxigenic from non-aflatoxigenic strains of *A. flavus*. This achievement is crucial to minimize fungal contamination of food, feed, and agricultural commodities, thereby reducing the risk of subsequent aflatoxin consumption.

## Introduction

Aflatoxins are highly toxic secondary metabolites that emanate mainly from filamentous fungi *Aspergillus flavus* and *Aspergillus parasiticus* in agricultural and food products [ 1 , 2 ]. These secondary metabolites constitute the etiology of a wide range of carcinogenic, mutagenic, and deleterious complications in humans and various animals [ 3 , 4 ]. There are at least 20 known fungal metabolites, 14 of which are primarily studied as typical aflatoxins. Only six of them, namely aflatoxins B_1_, B_2_, G_1_, G_2_, M_1_, and M_2_, can be found typically in food [ 5 , 6 ]. Aflatoxin B_1_ (AFB_1_), the most toxic one, is produced by *A. flavus* and *A. parasiticus*. The liver is the main target of aflatoxins. The consumption of contaminated food over an extended period of time may lead to adverse consequences for the liver, including damage to hepatic tissues and cells that become visible or remain microscopic. AFB_1_ is shown as a significant immunosuppressive, hepatotoxic, teratogenic, and mutagenic agent [ 2 , 5 ].

Approximately 30 genes are thought to be involved in the biosynthesis of aflatoxins. The number of genes and corresponding enzymes has reportedly increased over time. Aflatoxin pathway genes in *A. flavus* and *A. parasiticus* are clustered within a 75-kbp. The DNA sequence containing the 25 genes or open reading frames represents a well-defined aflatoxin pathway gene cluster [ 6 - 8 ]. Since the discovery of aflatoxins, extensive and costly efforts have been made to identify and monitor the presence of aflatoxins in food products, while primarily aiming to establish strategies for their control [ 9 - 11 ]. To detect aflatoxins, several highly accurate methods have been developed, including high-performance liquid chromatography (HPLC), gas chromatography-mass spectrometry, and immunoassay-based assays. Meanwhile, usually, detection of aflatoxin-producing fungi depends on isolation protocols and culture methods which are both time-consuming and require taxonomic expertise [ 4 , 12 , 13 ]. 

A group-specific assay was described for the detection of aflatoxin-producing species by monitoring several aflatoxin biosynthesis genes, including aflatoxin regulatory (*aflR*), o-methyl transferase (*omt*), norsolorinic acid reductase (*nor1*) and versicolorin A dehydrogenase (*ver-1*) [ 14 , 17 - 19 ]. Both systems revealed an expected triplet pattern for aflatoxigenic strains of *A. flavus* and *A. parasiticus*, indicating the presence of all three targeted biosynthetic genes in these strains. According to the literature, about 40-50% of isolated *A. flavus* strains produce aflatoxin. This inability to synthesize aflatoxin could be a result of gene deletions or other mutations in aflatoxin biosynthetic genes [ 6 , 8 ]. 

Usefulness of PCR methods to monitor the quality and safety of agricultural commodities can be well exploited to differentiate the toxigenic and non-toxigenic strains [ 17 , 18 ]. Several multiplex PCR systems have been developed recently for the detection of aflatoxin-producing fungi [ 14 - 20 ]. The multiplex PCR is a rapid method for identifying and monitoring aflatoxigenicity in *A. flavus* which employs a combination of primer sets to generate amplicons of various sizes that are specific for various DNA sequences. 

In the present study, multiplex PCR, as a genome-based method, was used for the detection of aflatoxin-producing *A. flavus* strains isolated from pistachio orchard soil for the first time in Iran. Two chromatographic techniques, namely thin layer chromatography (TLC) and HPLC were used for the qualitative and quantitative assay of AFB_1_ produced by examined strains. 

## Materials and Methods

### Fungal strains and cultures

The present study was performed on *A. flavus* PTCC5004, (AFB_1_-producing standard strain) and 25 isolates of *A. flavus* isolated from soils of pistachio orchards obtained from the Pathogenic Fungi Culture Collection of the Pasteur Institute of Iran [ 13 ]. All fungal strains were cultured on sabouraud dextrose agar (SDA, Merck, Germany) and kept at 28 °C for 4-5 days. The fungal spores were collected using a sterile tween 80 solution, 0.1% (v/v), washed three times, and counted. The 5×10^3^ spore/ml were cultured in 100 ml erlenmeyer flasks containing 25 ml YES (yeast extract 2% and sucrose 15%) broth in an orbital shaker (150 rpm) for 3 days at 28 °C. Fungal mycelia were separated through Whatman No.1 filter paper. Each fungal mycelia were washed three times with sterilized and deionized water and kept at -80 °C for the next DNA extraction step. The filtrated cultures were stored at -20 °C for AFB_1_ determination [ 13 ]. 

### Qualitative measurement of Aflatoxin B_1_ by thin-layer chromatography

For qualitative measurement of AFB_1_ by TLC technique, 10 ml of the YES medium form 3 days fungal culture were mixed with 10 ml of chloroform (Merck, Germany). The chloroformic extracts were concentrated by a rotary evaporator (EYELA N-1000, Japan) until dry. The residue was dissolved in 1 ml methanol (Merck, Germany), and 5 µl of samples were spotted on silica gel 60 F254 plates (E. Merck, Germany) comparing AFB_1_ standard (1 ρg/ml). The plates were developed with a mixture of chloroform and methanol (98:2 v/v) and visualized under UV light (365 nm). The intensity and Rf values of the spots were compared to those of the AFB_1_ standard that appears as blue fluorescent spots [ 21 ].

### Quantitative measurement of Aflatoxin B_1_ by high‐performance liquid chromatography

Quantitation of AFB_1_ was carried out using HPLC (KNAUER D-14163 UV-VIS system, Germany). Briefly, 50 µl of each of the chloroformic extracts sample was injected into the HPLC C18 reverse phase column (TSKgel ODS-80TS; 4.6 mm ID × 150 mm, Tosoh Bioscience, Japan) and eluted at a flow rate of 1 ml/min by water-acetonitrile-methanol (60:25:15, v/v/v). The amount of AFB_1_ was measured at a wavelength of 365 nm using a fluorescence detector. A standard curve of AFB_1_ was plotted using various concentrations of AFB_1_ standard (1, 5, and 10 mg), and the amounts of AFB_1_ in unknown samples were calculated by comparison of the undercurved areas of the samples with authentic standards. The elution time of the samples was compared with pure AFB_1_ retention time (12.25 min) and quantified based on the ratio of the peak area of samples to those of the standards [ 21 ].

### DNA extraction and qualification

Total DNA was extracted from three-day-old mycelia grown on YES as described above. Fungal mycelia were thoroughly ground in a porcelain mortar and 10-50 mg homogeneity of the fungus was poured into a 1.5 ml tube. The DNA was extracted with a commercial kit from Qiagen (QIAamp DNA MiniKit; Qiagen, Germany) according to the instructions of the manufacturer. Afterward, 300 μl of lysis buffer (100 mM Tris, pH 8, 10 mM ethylene-diamine-tetraacetic acid [EDTA], 100 mM NaCl, 1% sodium dodecyl sulfate, and 1% Triton X-100) was mixed with 300 μl phenol-chloroform, vortexed, and centrifuged at 5, 000×g for 5 min. Isopropanol was added to the supernatant at the same volume, and the tube was incubated at -20 °C for 20 min followed by centrifugation at 12, 000×g for 10 min. The precipitate was washed with cold 70% ethanol, dried, and dissolved in 50 μL of TE buffer (10 mM Tris, 1 mM EDTA). To ensure DNA extraction, 5 μl of the extracted solution was electrophoresed on 1% agarose gel. Presence of a band at the top of the gel indicated the presence of DNA. Its optical density was determined at 260 and 280 nm [ 22 ].

### Primer design

Reliable sequences of several molecular identification targets were downloaded from the National Center for Biotechnology Information (https://www.ncbi.nlm.nih.gov/pubmed/) (Table 1). They included one regulatory (*aflR*) and three structural (*omtA*, *omtB*, and *ver-1*) genes in the aflatoxin gene cluster of *A. flavus* which were targeted for aflatoxin production [ 17 ]. Primer design for multiplex PCR application was performed by Ultiplex software (http://ultiplex.igenebook.cn), assessing critical factors, such as compatibility of the primers. It should also be noted that the production of additional bands or spurious hybridizations of primer pairs to each other in amplification reactions was avoided. The oligonucleotide primers were synthesized by Nedayefan Co., Iran.

**Table 1:** Multiplex-polymerase chain reaction banding patterns and Aflatoxin B_1_ production profile in isolated *Aspergillus flavus* strains

*Aspergillus flavus* strains	AFB_1_ assay (ng/mg fungal dried weight)	AFB_1_ detection by TLC	*omtA*	*omtB*	*ver-1*	*aflR*
*A. flavus* 255	41.2±0.2	+	+	+	+	+
*A. flavus* 267	75.7±11.8	+	+	+	+	+
*A. flavus* 137	321.6±55.0	+	+	+	+	+
*A. flavus* 191	33.1±0.6	+	+	+	+	+
*A. flavus* 193	72.7±0.5	+	+	+	+	+
*A. flavus* 288	51.6±0.3	+	+	+	+	+
*A. flavus* 116	31.8±8.1	+	+	+	+	+
*A. flavus* 186	29.5±0.5	+	+	+	+	+
*A. flavus* 309	40.4±10.2	+	+	+	+	+
*A. flavus* 285	1.2±0.2	+/-	-	+	+	+
*A. flavus* 83	0.0	-	+	+	+	-
*A. flavus* 86	0.0	-	+	+	+	-
*A. flavus* 101	108.6±14.0	+	+	+	+	+
*A. flavus* 209	140.6±14.7	+	+	+	+	+
*A. flavus* 244	2.2±0.4	+/-	-	+	+	+
*A. flavus* 224	19.1±0.3	+	+	+	+	+
*A. flavus* 165	122.6±0.7	+	+	+	+	+
*A. flavus* 154	0.0	-	-	+	+	-
*A. flavus* 213	44.1±1.2	+	+	+	+	+
*A. flavus* 313	14.0±3.0	+	+	+	+	+
*A. flavus* 417	45.0±3.0	+	+	+	+	+
*A. flavus* 425	2.5±0.2	+/-	-	+	+	+
*A. flavus* 472	36.0±2.0	+	+	+	+	+
*A. flavus* 488	2.0±0.5	+/-	-	+	+	+
*A. flavus* 494	55.6+3.0	+	+	+	+	+
*A. flavus* 5004	255.6±10.2	+	+	+	+	+

TLC: thin layer chromatography, AFB1: Aflatoxin B1, aflR: aflatoxin regulatory, omt: o-methyl transferase, ver-1: versicolorin A dehydrogenase

### Multiplex polymerase chain reaction

Multiplex PCR amplification was set up and performed on the DNA extracted from all fungal isolates under the following thermal conditions: 5 min at 95 °C followed by 35 cycles of 30 s at 95 °C, 30 s at 65 °C, and 30 s at 72 °C and a final extension step for 2 min at 72 °C [ 17 , 19 ]. The reaction mixture contained 7.5 µl of 2× PCR master mix (Ampliqon, Denmark), 10 pmol of each primer, and 2 µl of DNA template, and reached a total volume of 15 µl by distilled water. The positive and negative controls were included in each amplification reaction. The PCR products were placed into loading STAR (Dyne Bio, Korea) for electrophoresis in 1× Tris-acetate-EDTA buffer on 2.0% agarose gel. Finally, the PCR product banding patterns were analyzed and compared to a DNA ladder (100 bp) (Table 2).

**Table 2:** Sequence of primers and size of amplicons

Primer	Sequence [5'- 3']	Amplicon size (bp)	References
*omtB-F*	GCCTTGACATGGAAACCATC	1333	[ 17 ]
*omtB-R*	CCAAGATGGCCTGCTCTTTA		
*aflR-F*	TATCTCCCCCCGGGCATCTCCCGG	1032	[ 17 ]
*aflR-R*	CCGTCAGACAGCCACTGGACACGG		
*ver-1-F*	ATGTCGGATAATCACCGTTTAGATGGC	895	[ 17 ]
*ver-1-R*	CGAAAAGCGCCACCATCCACCCCAATG		
*omtA-F*	GTGGACGGACCTAGTCCGACATCAC	797	[ 17 ]
*omtA-R*	GTCGGCGCCACGCACTGGGTTGGGG		

### Statistical analysis

Descriptive statistics were performed in GraphPad Prism software (ver. 9.5.0.). Analysis of variance test was used as required to compare categorical variables. The *P*-values of less than 0.05 were considered statistically significant.

## Results

### Aflatoxin B_1_ production assay

The results of the HPLC analysis regarding the mean level of AFB_1_ produced by toxigenic isolates on YES broth are summarized in Table 1. Comparison of AFB_1_ production of *A. flavus* strains showed that the range of toxin production was between 0-321.6 (ng/mg fungal dried weight). *Aspergillus flavus* strains were classified into four groups according to their AFB_1_ toxin production, including non-producers, low producers, intermediate producers, and high producers. The strains that produced no AFB_1_ were assigned to Group A, low producer strains that produced between 0.1 and 10 ng/mg AFB_1_ were assigned to Group B, intermediate producer strains that produced between 10.1 and 100 ng/mg AFB_1_ were classified as Group C, and high producer strains that produced more than 100 ng/mg AFB_1_ were categorized as Group D. Except for samples 83, 86, and 154, all the other tested strains were positive for aflatoxin production in TLC analysis. This finding was validated by the HPLC method and the values were determined precisely (Table 1).

### Multiplex polymerase chain reaction

**Figure 1:**
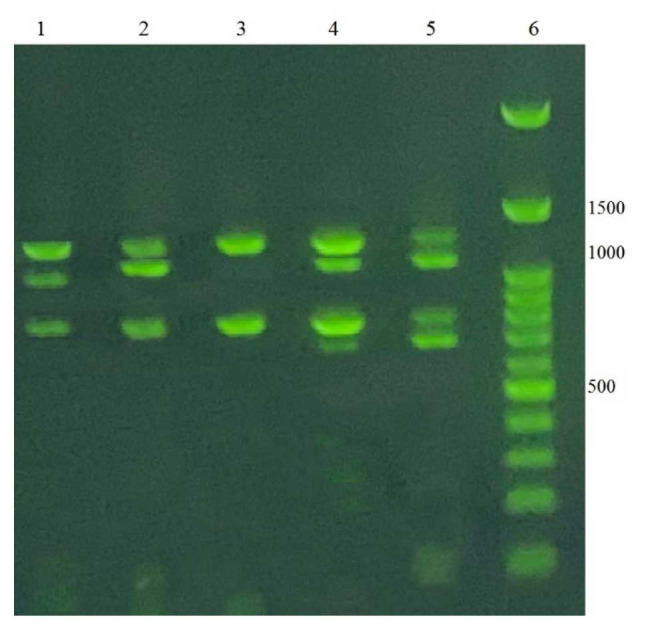
Detection of *Aspergillus flavus* isolates producing aflatoxin B_1_ using the multiplex-polymerase chain reaction method. Lane 1: *A. flavus* 425, Lane 2: *A. flavus* 488, Lane 3: *A. flavus* 154, Lane 4: *A. flavus* 186, Lane 5: *A. flavus* 5004, Lane 6: Ladder (100 bp).

The Multiplex PCR method yielded successful results in the analysis of the primers of *omtA*, *omtB*, *ver-1*, and *aflR* genes involved in the aflatoxin biosynthetic pathway. According to the multiplex PCR results, aflatoxin-producing *A. flavus* strains isolated from pistachio orchards soil performed all four bands in both sets of primers. Generally, 18 out of the 25 *A. flavus* (72%) demonstrated all four primer-related bands in moderate and high aflatoxin-producing strains (Figure 1). 

However, in four low toxin-producing *A. flavus* strains (16%) (244, 285, 425, and 488), despite the presence of the toxin being detected by HPLC, one of *omtA*, or *aflR* band did not present in the multiplex PCR. Moreover, the non-aflatoxin-producing *A. flavus* strains were incapable of generating one or two bands belonging to *omtA* or *aflR* (Figure 1). 

**Figure 2:**
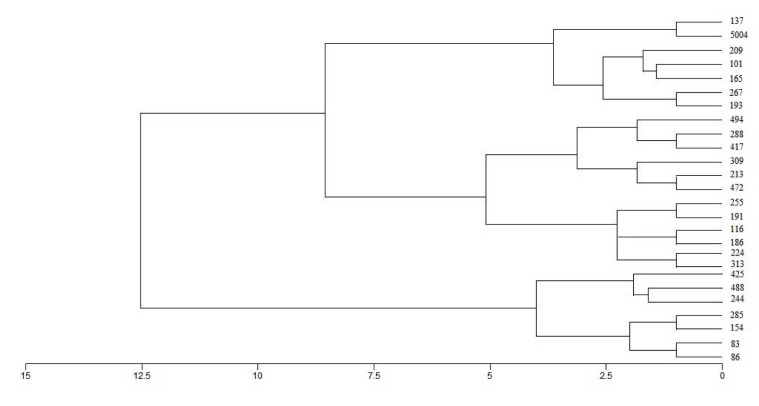
Dendrogram using multiplex polymerase chain reaction bands and the resultant classification between the binding patterns categories and toxin production.

Positive and negative controls were used for the assessment of the precision and accuracy of the respective methods. In the three non-aflatoxin-producing *A. flavus* strains (83, 86, and 154) (12%), the results showed that the band belonged to *aflR* as a regulatory gene was absent in strain number 83 and 86 although both *omtA* and *aflR* bands were not present in *A. flavus* 154. Patterns of the resultant bands can be classified into three broad categories (Figure 2).

## Discussion

As aflatoxins are generally known to have toxic, carcinogenic, mutagenic potential activities, there is an urgent need to develop rapid and highly specific detection methods that are capable of precisely differentiating non-toxigenic from toxigenic strains with a reasonable duration [ 2 , 5 ]. The analytical methods, such as TLC and HPLC, are considered gold standard methods and therefore, have been assigned the most amount in various research projects [ 4 , 5 ]. However, these advanced physicochemical methods have some disadvantages. For instance, they are time-consuming for extensive preparation of samples and need very expensive equipment and an expert force that can measure the amount of toxins with these two methods since not every laboratory is able to use these two methods. 

Detection of aflatoxigenic *Aspergilli* strains using PCR was reportedly described for monitoring food and feed quality and safety [ 12 - 16 ]. Multiplex PCR is used for simultaneous amplification of multiple targets/loci and is applied as a diagnostic tool to detect multiple gene mutations [ 23 ]. The mentioned limitations made us investigate the possibility of using the multiplex PCR method as an alternative to these two reference methods to differentiate our isolated toxigenic and non-toxigenic *A. flavus* strains. 

In this study, the multiplex PCR method was used for the first time to monitor aflatoxin-producing ability in *A. flavus* strains isolated from pistachio orchard soil by analysis of the four aflatoxin biosynthetic pathway genes, namely *aflR*, *omtB*, *omtA*, and *ver-1*. The *aflR* and *omt* gene primers were used since *aflR* gene regulates the expression of *omt* gene, a structural gene enclosed in the aflatoxin (AF) biosynthetic pathway, and *omt* gene is necessary for almost the final formalities of AF biosynthesis [ 7 - 9 ]. The presence of these two genes clearly indicates the aflatoxin production machinery and the detection of aflatoxigenic strains with bands corresponding to *aflR* and *omt* genes confirms the identity of the invading genus [ 17 , 23 ].

According to the determination of AFB_1_ production using TLC and HPLC methods, *A. flavus* strains were divided into four categories, namely high, moderate, low, and non-toxin producers. The multiplex PCR assay results showed that AFB_1_-producing *A. flavus* strains revealed all four bands (1333, 1032, 895, and 797 bp) in both sets of primers and non-toxic strains failed to produce at least one of the four bands. In AFB_1_ non-producer *A. flavus* strains 83 and 86, the band of *aflR* as the regulatory gene was not observed. Moreover, the band that represented the *omtA* gene was missing in AFB_1_ non-producer *A. flavus* strain 154 and low producer *A. flavus* strain 244 could be attributed to the mutation in *omtA* gene. 

Among the 22 positive strains that were analyzed using HPLC and TLC methods, all four multiplex bands were clearly visible in 18 strains. Nevertheless, regarding 4 out of the 22 strains, one of *omtA* or *aflR* bands or both of them were not present in the multiplex PCR. However, in some non-toxigenic and low toxin-producing *A. flavus* strains, the band that represented the *omt*-A and *aflR* genes was missing, whereas all four bands were observed in moderate and high toxigenic *A. flavus* strains. *Aspergillus* flavus strains, which were incapable of producing toxins, had a mutation in at least one of the relevant genes, thereby causing the absence of at least one band in the Multiplex PCR. The absence of bands that indicated no AFB_1_ production by some strains can be considered in line with previous findings of corroborative indications where a lack of aflatoxin biosynthesis occurred occasionally due to genetic mutations.

In similar studies, the Multiplex PCR method was used for the detection of the AFB_1_ toxin in various agricultural and horticultural products, including Meju and Korean fermented foods and seeds [ 17 ]. Application of the multiplex PCR method for the detection of aflatoxin in Meju showed an increase in the accuracy and speed of aflatoxin detection. Latha et al. in their study showed that *aflR*, *nor-1*, *ver-1*, and *omt4* primer pairs gave specific multiplex PCR amplification and could be used as a marker to clearly differentiate between the aflatoxin-producing and non-aflatoxigenic *Aspergilli* [ 23 ]. In similar research, Criseo et al. studied the differences between aflatoxin-producing and non-producing *A. flavus* group [ 19 ]. They obtained a four-band pattern for all producers and a variable pattern for non-producers of aflatoxin and suggested that the lack of aflatoxin production apparently does not need to be related to an incomplete pattern obtained in quadruplex PCR. It can be concluded that different types of mutations may be responsible for the inactivation of the AF biosynthetic pathway genes in other *A. flavus* strains as was indicated by the results of the present study [ 23 ]. 

The non-toxigenic *A. flavus* strains showed variable results in the PCR assay. *Aspergillus flavus* BFE310 was negative in all three PCR assays, indicating a deletion of the whole or a major part of the aflatoxin biosynthetic gene cluster [ 15 ]. Specifically, *A. flavus* BFE311 showed negative results for the *omtA* gene primer pair, indicating a deletion in this gene. However, *A. flavus* BFE301 tested positive for the complete triplet pattern, indicating another type of mutation, possibly in a regulatory gene. It seems that all genes involved in aflatoxin production are clustered together, and the deletion of one gene would almost certainly disrupt aflatoxin biosynthesis [ 7 , 8 , 16 ]. 

Five genes, including, *aflR*, *aflS*, *aflD*, *aflO*, and *aflQ* were determined from various aflatoxigenic strains of *A. flavus*. This protocol shows a good correlation between the expression patterns of AF biosynthetic pathway genes, analyzed by multiplex reverse transcription-PCR (RT-PCR) and aflatoxin production [ 20 ]. False-positive results are obtained from food samples when several non-aflatoxigenic *Aspergillus* strains have distinct target gene deletion patterns in the same pistachio sample [ 20 ]. When screening pistachios for aflatoxigenic fungi for diagnostic purposes, false-positive results are usually regarded as less troublesome than false-negative results. The latter may have severe consequences for human health. In this regard, a crucial suggestion is to incorporate a control strategy into a future study and develop ways to ensure the proper interpretation of PCR results, while also enhancing the sensitivity of the Multiplex PCR method in the assessment of heterogeneous food samples [ 23 - 25 ]. 

Expressions of four AF biosynthetic pathway genes (*aflD*, *aflO*, *aflP*, and *aflQ*) were evaluated in 24 *A. flavus* strains by RT-PCR [ 21 ]. The results showed that transcription of the four genes was not always correlated with AF production, but there was a relationship between higher amounts of AFB_1_ production and expression of *aflO* and *aflQ* [ 21 ]. Rashmi et al. evaluated multiplex PCR assay for the concurrent detection of four major mycotoxin metabolic pathway genes, viz. *nor1* (aflatoxin), *Tri6* (trichothecene), *FUM13* (fumonisin), and *otanps* (ochratoxin A) using 10 reference strains [ 25 ]. The concluded mPCR results were further evaluated with HPLC, and in general, both methods provided unequivocal results. 

The five targeted AF cluster genes, namely *aflD*, *aflR*, *aflS*, *aflM*, and *aflP* were used for the examination of 15 aflatoxigenic isolates of *A. flavus* that were obtained from dairy feeds in Zimbabwe. Two genes, *aflD* and *aflS*, were significantly associated with aflatoxigenic *A. flavus* isolates [ 26 ]. The mycotoxigenic fungi belonging to various genera of *Aspergillus* spp., *Alternaria* spp., *Penicillium* spp., and *Fusarium* spp. were determined based on the eleven-primer pairs cocktail using multiplex PCR [ 27 ].

## Conclusion

Detection of mycotoxin-producing fungi in an early stage is crucial for controlling mycotoxins from entering the food chain. Results of the present study indicated that multiplex PCR can be used as an accurate method for the detection of *A. flavus* strains that produce AFB_1_ in pistachio soil. Detection of aflatoxin biosynthesis *nor1*, *ver-1*, *omtA*, and *aflR* genes by multiplex PCR led to results that correlated significantly with the measurement of aflatoxin production by TLC and HPLC methods. The multiplex PCR assay could be a useful strategy to overcome the major problems of conventional mycotoxin analytical techniques, such as TLC and HPLC, with the aim of high‐throughput monitoring of major aflatoxin‐producing fungi during the processing steps of food and feed commodities.
